# Information from physicians and retention of information by patients – Obstacles to the awareness of patients of progressing disease when life is near the end

**DOI:** 10.1186/1472-684X-7-2

**Published:** 2008-02-28

**Authors:** Lena Hoff, Göran Hermerén

**Affiliations:** 1 Department of Medical Ethics, Lund University, Sweden

## Abstract

**Background:**

Discrepancies between the information that patients have received and the patients' awareness of their condition have frequently been observed in literature and given a number of different explanations. The chief contribution of this study is that by following patients over time it is possible not only to notice any changes in the patients' knowledge or awareness of their disease, but also to investigate the interview material for possible reasons for those changes. Since the study is based on two different groups of patients it will also be possible to notice if the category of disease matters for patients' awareness of their condition.

**Methods:**

Twelve patients with malign haematological diseases or lung cancer were followed with interviews from diagnosis to cure or death, or at most for two years. The method is qualitative. Semi-structured interviews were conducted, transcribed into written text, and then used for a qualitative content analysis.

**Results:**

During the process of analysis four different expressions (subcategories) emerged about the awareness of patients concerning their health status: informed and aware, not informed and not aware, aware though not informed, or not aware though informed. Then the search started for obstacles to the awareness of patients regarding their progressing disease and approaching death. Four kinds of obstacles were found: due to the physician, the patient, the physician and the patient in collusion, or neither to the physician nor the patient but the insidious way in which lung cancer (mostly) and haematological malignancies (occasionally) progress.

**Conclusion:**

To optimize the care of patients who wish to be informed and aware during their disease, it is important that the health care staff recognizes potential obstacles to the awareness of patients in order to minimize such obstacles. The physicians could improve their communication with patients with life-threatening diseases, and avoid having a narrow focus on the treatment calendar. The patients could be encouraged to have a more proactive attitude in their communication with their physician.

## Background

The purpose of this study was to draw attention to probable discrepancies between the information the patients had received from their physician and the awareness patients had of their condition in all phases of their disease, from diagnosis until their health of state was terminal. Even when patients had been informed by their physician, they sometimes seemed to lack awareness of their disease. Many explanations have been proposed for this phenomenon. The physicians fail to address the need of the patients to be informed [1]. The patients' abilities to receive information are affected by underlying mistaken beliefs [2]. The language used by medical professionals does not always have the same meaning to laymen as to themselves [3]. Between the physician and the patient, collusion may be present, with the physician avoiding talk about death and the patient gratefully accepting every opportunity to "forget" what is coming [4]. It has also been emphasized that such collusion might be both what the patient wants and what the patient needs [5]. The process of information is also complicated, demanding both intellectual and psychological understanding of patients [6]. Finally, the patients have different abilities to cope with and integrate the bad news into their life [7].

All studies referred to above are close to this one in different ways. The chief contribution of this study is that, by following a group of patients over time, it is possible not only to notice any changes in the patients' knowledge and awareness of their disease but also to investigate the interview material for reasons for those changes.

As the study is based on two different groups of patients, diagnosed with either malign haematological or non-operable lung cancer, it should also be possible to notice if the category of patients of this study matters for their knowledge and/or awareness of their condition.

Before going further, we need to clarify the use in this study of the two terms in focus: "information" and "awareness", as they are both burdened with some difficulties. In this study it is the patients' view of information that we have chosen.  The term information is used only to denote information given to the  patients by their physician. We have listened to the words of patients. If they said that they had been informed, they were documented as informed. If they said that they had not been informed, they were documented as uninformed. The term awareness is used to acknowledge what patients know in all phases of their disease, regarding their actual condition of health, of progressing disease and of approaching death. Mostly, the information received constitutes the basis for the patients' awareness of their health status. Nevertheless, it is quite possible that a patient had been informed but for various reasons acted as if no information has been received. Moreover, to be aware is not a question of either-or, but of being more or less aware. A patient might be informed and aware of his or her condition. It might also be possible to find a patient who is informed, yet not aware of his or her state of health, or that the patient is aware even though he or she is not informed.

### The aim of the study

The aim of the study was to follow a small group of severely ill patients with interviews from diagnosis to cure or death, or at most for two years, to study the patients' view of information compared to their awareness of their disease. The first step was to search through the interview material to notice any changes in the patients' awareness of their disease. The next step was to search for probable explanations for such changes. Since this is a qualitative study the purpose is to describe, interpret and clarify differences in the awareness of patients during the trajectory of their disease to obtain a deeper understanding, and not to make statistical generalizations. The purpose of the paper is not to generalize beyond the material in the paper. No statistical frequencies are stated in the conclusions.

## Methods

The materials and methods of this study are the same that were used in a previous study of ours (Hoff, et al 2007). These studies have both been based on the recurrent interviews with 12 seriously ill patients, 7 patients with malign haematological diseases (acute lymphocytic leukaemia, acute myelocytic leukaemia, myeloma) and 5 patients with non-operable lung cancer diseases (adenocarcinoma and squamous cell cancer). The patients with acute leukaemia were to be treated curatively, the patients with lung cancer only in a palliative way. The treatment of the later was essentially about slowing the progress of the cancer, or if possible to stop it, while the patients with acute leukaemia were expected to have a real chance of recovery. The decision to include these two categories of patients in the study was, however, due to the similarities between them. The outbreak of the disease often comes all of a sudden to both categories of patients. All patients were to be treated with intensive cytostatic regimes. Without treatment they all would have been dead within a few months. The treatment is estimated to be effective in most cases, but for many patients the malignancy will be back after some time. The survival rates are therefore low for both categories of patients. Only 20% of patients diagnosed with acute leukaemia are estimated to be alive two years later [8]. Some patients with myeloma get an aggressive form of the disease bringing them to death within a year, while others might live with good quality of life for several more years to come [9]. Among patients with non-small-cell lung cancer only 3–7% are estimated to be alive five years later, while 50% die within seven months [10].

The patients were diagnosed at Örebro University Hospital, Sweden, and they were consecutively asked by their physician to join the study. The patients were given both oral and written information (patient letter) by their physician. In the patient letter it was stressed that participation was voluntary and that the patients could withdraw whenever they wanted without any negative consequences for their treatment. A summary description of the participants and the number of interviews is presented in Table 1. The letters A-G in the table and in the following refer to patients with haematological diseases and the letters V-Z to patients with lung cancer.

**Table 1:** Study participants and number of interviews

Category of patient	No of Interviews	Sex	Age
7 Hematologic	44	3 men/4 women	37–80 yrs old
5 Lung cancer	44	2 men/3 women	60–71 yrs old

Out of 11 patients with acute leukaemia or myeloma 10 were asked to participate and 7 of them accepted. Of 9 patients with lung cancer, 7 were asked and 5 accepted. Exclusion criteria were if the patient was unable to give informed consent or understand the questions. The interview period was 2002–2005. The settings were either the hospital or the patient's home. The total number of interviews was 88, lasting 5–90 minutes each, depending on the current health status of the patient. For the distribution of interviews, see Table 2.

**Table 2:** The distribution of interviews

Patient	Diagnosis/Treatment 1	Treatment 2	Terminally	Totally
A	3	-	-	3
B	1	-	-	1
C	9	2	2	13
D	3	-	-	3
E	6	3	1	10
F	3	-	-	3
G	11	-	-	11
V	2	-	1	3
W	6	-	1	7
X	11	2	1	14
Y	8	2	1	11
Z	8	-	1	9
				**88**

The very first interview followed the interview-guide, see Additional file 1. Depending on what happened to the patient new questions emerged, see Additional file 2. The intention was neither to interview the patients as soon as possible after they had received their diagnosis, nor to follow the patients during the whole processes of their dying. The first interview was held from six days to six weeks after the diagnosis had been given to the patients. All patients were then expected to have overcome their very first shocking phase. The interviews came to an end as soon as the patients said that they were about to die, since further treatment would not stop the progress of the disease.

For closer information on differences in the distribution of interviews, ethical considerations, collection of data, we would like to refer to the above-mentioned previous study of ours [11].

### Method of Analysis

Inspired by an approach used in nursing ethics [12], the interviews transcribed into written text were used for a qualitative content analysis. Firstly, all typed material was read through several times; secondly, all material not relevant to the purpose of this study was excluded. The remaining material was then divided into three domains of investigation: the disclosure of the diagnosis and information during the first treatment, information during the second treatment, and information when in terminal phase. These domains were carefully searched through in order to establish meaning units and condensed meaning units. Then the process of coding started with the labelling and summing up the text passages, all followed and checked by a co-reader. The codes related to the questions of knowledge and awareness of the patients were gathered and then compared to each other. During this process of analysis four different expressions (subcategories) emerged about the awareness of patients regarding their condition: the patients as informed and aware, as not informed and not aware, as aware though not informed and as not aware though informed. These four different expressions were then used as categorization of the awareness of the few patients included in the study.

The next step of the analysis started the investigation of the latent content of categories, the search for obstacles to the awareness of patients regarding their progressing disease and when life came close to an end. Four kinds of obstacles were found: due to the physician, the patient, both the physician and the patient or to neither of them.

## Results

### Four categories of awareness of patients regarding their state of health

The categorizations of patients were not static, however. With new information they changed, as the disease developed. Initially, all patients declared that they had been informed in an open and straight way about diagnosis, treatment plans, possible side effects, as well as of prognosis. Thus far, they were all categorized as "informed and aware". However, to patient V with lung cancer the disease was progressing. In spite of treatment the cancer was there. This she was not informed about. In fact, she said, she had to plead for information, before she got to know the sad news. She was now categorized as "not informed" but when knowing later she was re-categorized as "informed and aware". The remaining four patients with lung cancer had a sudden and remarkable recovery, shortly after the treatment had started. Now, feeling well, the threat of death seemed to be much further away. "There is more of a hope, I think" (Y: 2). Only one patient, and only once more, talked about the incurability of the disease (X: 8). The categorization of these patients then changed somewhat from "informed and aware" to "not aware, though informed".

When the disease recurred it affected the awareness of patients differently, depending on the category of disease. For patients with leukaemia, the neutrophile leukocyte test brought about the first alarm that the malignancy might be back. Then a bone marrow test was taken to confirm it, or not, and the test result was given to the patient (C: 10, E: 9). These patients therefore remained categorized as "informed and aware". For the patients with lung cancer the return of cancer seemed to have been hidden behind different diseases, such as thrombosis (Z), recurrent pneumonia and dyspnoea (Y, X) colds and high fever (W). Regular tests were taken for these diseases and the test results were continually given to the patients. Nevertheless, they seemed little by little to have lost their knowledge of their disease. The revival of cancer did not seem to have become obvious to them until their physician suggested a second regime of cytostatics (Y: 9), or until the phase was terminal (X, Z, W). These patients were categorized as "not aware, though informed".

In terminal phase the patients' awareness differed again. Two of the patients (C) and (V) received the information verbally from their physician that the treatment had failed and that another treatment was considered futile. They therefore remained "informed and aware", even though patient V again had to ask before she got the information (V: 3). Patient E became aware of the transition to the terminal phase, not because of information from her physician but because of the changing values of all tests taken. During all the time of her disease she had been following all test results most carefully. This second time the test values of haemoglobin, thrombocytes, neutrophile leukocytes changed, patient E understood what it all was about. Now, she expected a bone marrow test, as before, to be taken to confirm her state of health as terminal. However, this time her physician withheld the test. Then there was no confirmatory test result to convey to the patient, who nevertheless understood her state as terminal. Therefore she was categorized as "aware, though not informed". To patients with lung cancer the X-rays did not reveal the cancer to be in progress and death to be close until the last weeks of their lives. Whenever the cancer turned up in a manifest way in the tests taken, the patients of both categories were informed, and categorized as "informed and aware". So was patient X, even though she declared that she had not verbally been told that she was in a terminal phase. Yet, she had understood, she said: "I do not think he (the physician) has told me so very straight, but he held my hand and so... so I did understand, what it was all about" (X:14). She was then categorized as "aware, even though not informed".

There was also patient Z who did not admit her phase to be terminal. She would soon be better, she said, two weeks before her death (Z: 9). Patient W declared himself to be all right only three days before his death. These two patients were therefore categorized as "not aware, though informed".

### Four categories of obstacles to the awareness of patients

#### 1) Obstacles due to the physician

Since the physicians are responsible for informing patients, one might think that the major reason for patients' lack of knowledge or awareness of their progressing disease must be reluctance on the part of the physicians to give patients bad news. But what we found, from the interviews with the patients, was that the physicians had been eager to inform the patients all results of tests taken. As tests were taken the patients were continually informed about the results. We even found some patients (D, F, and G) who complained about the amount of information given to them. "Actually, you can't keep it all in your head. It's too much" (F: 3). These patients wanted to be informed they said, but not about every test taken.

There was, as already noted, one patient who told the interviewer that she had had to ask for information, before she had got the bad news about the failure of treatment (V: 3).

There was also one finding of a physician withholding a potentially confirmatory bone marrow test (E: 10).

The patients' narratives also revealed that information from physicians was not only a matter of words. Mostly, the non-verbal communication seemed to have worked out positively as for patient X, as noted before. Patient V, on the other hand, told the interviewer how her physician had suddenly taken her hand, examined it closely, and then left her without a word. He had just left. The patient suspected that the physician had been looking for physical signs of her approaching death, but found it confusing that he had been examining her hand and not her cheeks (V: 3). Ill-functioning non-verbal information left the patient confused and worried.

Finally, all patients were asked whether they had talked with their physician about their approaching death or not. Only patient C said that he had. Patient V came to do it but not until she once again had asked for more information. Then she said: "Now, I quite often ask how long it will take before I die, but they are not able to answer! I find that very strange... I would have appreciated if there was some kind of statistics... I suppose there is, but they don't want to tell me" (V: 3). When she finally knew she added: "Maybe I shouldn't complain. Most of my questions might have been addressed now, but... still there are questions they have left without an answer... maybe, those questions they don't know how to answer, they just skip those" (V: 3). The interview material reflects a kind of reluctance on the part of the physicians to talk with the patients about their approaching death.

#### 2) Obstacles due to the coping strategies of patients

The patients developed different coping strategies. The words of some patients could easily have led to them being documented as not informed and/or not aware, and their coping strategy as an obstacle to their awareness. Through their answers to follow-up questions, they nevertheless appeared to be both informed and aware.

Some patients talked about their disease as if what was happening was not happening to them but to someone else, or that it was not happening at all. "You have to deceive yourself, you know, as if it is not happening to you, but to someone else" (X: 3). Patient A acted as if she did not know much about the danger of her disease. However, when asked if she knew of anyone who had got the disease acute leukaemia, she said she knew two people. They had both died (A: 2, A: 3). Patient W, all through his disease, simply turned his back on the whole question of his approaching death. In the last interview (three days before he died) we even talked about his 70th birthday which was to come three months later (W: 7). At the same time he commented for the first time on the presence of metastases in his body.

We also found that several patients were eager to find simple excuses when not feeling well. They could have been overstraining themselves, they said. "You see, I have had visitors coming Monday, Tuesday, Wednesday, Thursday, Friday... all week my workmates have been visiting me" (Z: 6). "It feels hard to notice that your strength is not the same as only a year ago. But I am a year older now, you know" (Y: 7). "Now we have finished the work with the new garage. Maybe that was too much?" (Y: 8). Patient X likewise blamed herself for having been too busy. "Yesterday, I was ironing. Probably, that was not very wise of me... the steam you know... and then I was out in my wheel chair for two whole hours, too much maybe... or maybe, I did catch a cold" (X: 8).

#### 3) Obstacles due to collusion between the patient and the physician

Collusions between the physician and patients have already been explored in a study by The et al, where it is stated that "both parties colluded in focusing on the treatment calendar and at the same time ignoring the long term". This is congruent with our finding. Of course, the patients with lung cancer noted that their health was declining in spite of recoveries from various attacks of pneumonia or infections. Yet, only seldom did these patients have a proactive attitude of asking, except for patient V as noted. Not even when the patients had sometimes prepared themselves for bad news did they ask for further information. "...patients seem to accept gratefully every opportunity offered by doctors to 'forget' the future" [13].

When patient Y was asked by the interviewer why he did not ask his physician about his approaching death, he explained: "The thing is, they can't really tell. There will always be facts that they don't know anything about" (Y: 10). "The physician hasn't got my timetable" (Y: 11). "I don't need to die from this. It could be from anything" (W: 5). "You know, even if I had not been ill I could be killed by a car or something" (X: 10). Another reason for not asking the physicians was given by patient G: "They [the physicians] are really sweet and so... but you don't want to put more burden on their shoulders" (G: 3). Another kind of collusion is illustrated by the patients who did not take much interest in the results of any test taken. They reckoned that they would be informed when there was something they ought to know about. "I'm not one of those who check up the test results. They'll tell me, I suppose, if there's something I should know" (G: 11).

#### 4) Obstacles due neither to the physician nor the patient, but to the insidious progression of the disease

One could have expected that the awareness of patients depended on the category of disease. The categories were chosen based on similarities between them, yet in some ways they differed from each other, as to the side effects of the cytostatic treatment of those patients. While patients with haematological diseases were in the course of treatment they often felt sick (nausea), they lost their hair, and they became hospitalized and strictly isolated. The cytostatic treatment of patients with lung cancer mostly did not affect them that hard. These differences in the side effects of the treatments did have an impact on the patients' awareness, but there were informed and aware patients with both categories of disease, and there were patients from both categories who were not informed and not aware during their disease. Even if most patients who were not informed and not aware were found among the patients with lung cancer, the demarcation line did not follow the category of disease, but the nature of the disease's progress. Whether the malignancy progressed gradually and insidiously or in a rapid and manifest way (confirmed in the test results), was decisive for the patients' awareness. This is the major finding of this study. As already noted, the revivals of lung cancer most often started hidden behind recurrent acute diseases, such as thrombosis (Z), recurrent pneumonia and dyspnoea (Y, X), colds and high fever (W). For these patients it took a long time until the progress of cancer was manifested and possible to identify by X-ray pictures. This is to be compared with patients with leukaemia suffering from infections and high fever. To them the neutrophile leukocyte test result was an immediate warning that the malignancy might be back. If this was the case, a bone marrow test was taken to confirm the return. However, to one of these patients with acute leukaemia the second relapse progressed much more gradually, much more like the relapses of patients with lung cancer.

Finally, there were the four out of five patients with lung cancer who, shortly after the cystostatic treatment had started, had a sudden and remarkable recovery making them feel so much better. It might not be correct to call this recovery an obstacle. However, the recovery contributed to a change of attitude in the patient. Now these patients were more hopeful. "Now, there is more of a hope, I think" (Y: 2). Feeling so much better there was no need for such a hurry, he said (Y: 3).

## Discussion

The starting point for us was the finding that all twelve patients in the study expressed a will to be well informed throughout the disease, which is congruent with the findings of many other studies [[14-16], and [17]]. If we take these statements by patients seriously, our aim ought to be to find out what could possibly be done to reduce the lack of knowledge and/or awareness of these patients. Improved disclosure and patients' right to obtain information about their condition is usually supported by the principle of autonomy, and non-disclosure by the principle of beneficence [18]. However, both practices can be ethically justified depending on the situation [19]. According to the Kantian way of thinking, autonomy is the basis for making a man able to act ethically as a person with responsibility. Autonomy is what is needed for man to remain human. For our purpose a less sophisticated interpretation of autonomy will do, according to which the normative value of information and awareness only exists when it is supported by the expressed will of patients or the goals of patients. Thus neither information nor awareness are intrinsic values [20]. If the patients instead had expressed an unwillingness to be informed, we then would have suggested that they should be less informed. But what we found was that the patients did want to know, but were not always informed or aware. What could then be done to reduce the lack of knowledge and/or awareness in patients?

### How to deal with the obstacles due to the physician

According to the interviews with the patients, the physicians seem to have been eager to inform them of all results of tests taken. Only one exception was found. Why there was a delay in the informing of patient V we do not know, we only know that she had to plead for information before she got the sad news, and that is what we question. Then there was the finding of a physician withholding a potentially confirmatory bone marrow test. Without a test there was of course no test result information to give the patient. However, withholding of a test might be ethically justified by the principle of non-malificence, that is, that the potential harm the test would cause the patient should not outweigh the benefit it would bring the patient. If you can not do any good, at least do no harm to the patient. However, questions arise how as to communicate this to the patient and how to prevent withholding of a test from making the patient (more than necessarily) less aware.

Some patients in the study regarded themselves as informed, albeit non-verbally. When the non-verbal communication worked properly, as for patient X, we have no ethical objections so far, as we think that the need of this patient was met. Unfortunately, this was not the case for patient V, and the question that arises is how to be cautious in the practising of non-verbal communication. Maybe the message understood without any words will function best as a predictor of what the physician has come to tell the patient [21].

When the communication concerned the end of life, silence became notable. The physicians seem to have left the hard questions without an answer. If the physicians do not provide space for such communication with the patients about their approaching death, who should?

### How to deal with the obstacles due to the patients

The patients developed different coping strategies. But their coping strategies (not even the strategy of denying) were not really found to be an obstacle to patients being aware of their health status. On the contrary, we found that the coping strategies all presupposed that information had been given to the patient, even though the information then was sometimes transformed into more bearable messages. According to psychological theories the coping strategy of denying functions as a defence, not to be removed or questioned [22,23].

There are patients who genuinely wish to have limited information, but their number is far smaller than many physicians assume. Good communication is required in order to be able to differentiate patients who need more information from those who do not want information.

### How to deal with the obstacles due to collusion between the physician and the patient

The collusions mostly consisted of focusing on the treatment calendar and at the same time ignoring the long-term perspective. To reduce the uncertainty of progressing disease and approaching death, could it be possible for the physicians to have a more proactive attitude (instead of waiting for the patients to ask for information)? On the other hand, could the patients be encouraged to be more proactive by asking?

The collusion was also of the kind that the patient could, more or less, leave it to the physician to decide what would be in the best interest of the patient. This could be ethically justified if this is in accordance with the expressed will of the patient. Otherwise, unless the patient is unconscious, such collusion is paternalistic and may be questioned.

### How to deal with the obstacles due to neither the physician nor the patient

Finally, we have come to what we found in this study to be **a major obstacle to patients' awareness of their disease**, namely the insidious way that lung cancer (mostly) and haematological malignancies (occasionally) progress in a slow and hidden way behind other diseases. In order to help these patients to become more aware during the trajectory of their disease, the question arises whether or not it would be possible to inform these patients what most probably is to come. If the physicians know, from experience with other patients, when the disease most probably is turning lethal, is there an obligation to inform the patient even before the malignancy is manifested in the test results? We believe so and once again we would like to refer to the principle of autonomy. It is the will of the patient that should be the determining factor. We believe in balancing the pros and cons for what seems best for each patient, even when the malignancy is not yet manifested in test results. Important for this standpoint is that the survival rates of patients with the diseases included in this study are low, as noted. The information would not have been that urgent, had the survival rates been higher. But as they are very low we believe that at least some of these patients would have gained from receiving more information about the fatal course of the disease.

How is the physician to inform the patient when the manifested test result is not yet at hand? The problem of communicating probabilities and uncertainties deserves to be explored, but this would lead us too far from the aims of this study and to do this more research is needed. It will have to be left for another study.

### Limitations, trustworthiness and validity

The limitation and trustworthiness of this study, as with other qualitative interview studies, are based on the small number of interviews. Besides, out of the twelve patients included in the study from start, only seven for various reasons were followed into the terminal phase (see Table 2). Patient B died four days after the first interview and three other patients (A, D, F) for various reasons were excluded from the study after three interviews each and we do not know what the awareness of those four patients would have been like in later phases of their disease. Nor do we know about patient G's awareness in later phases of the disease. She was followed for two years, but did not relapse during the period.

Another limitation was that the intention to follow the patients in a regulated way could not always be realized. Some prearranged interviews had to be shortened in time or postponed for days, or even weeks, because of the patient's current state of health.

Finally, even if the intention was to follow the words of the patients as closely as possible, there was always the possibility of misinterpretations or over-interpretations. To limit such risks confirmatory questions were used, the process of coding followed and checked by a co-reader, and critical researchers, physicians and co-readers have been invited to continuously read and check the interpretations made. According to S. Kvale, valid knowledge emerges where the consistency and internal logic of statements as well as the research procedure is tested: "valid knowledge claims emerge as conflicting interpretations and action possibilities are discussed and negotiated among the members of a community" [24].

## Conclusion

The results of this study indicated four kinds of obstacles to the awareness of patients of their progressing disease. To optimize care of patients who wish to be informed and aware during their disease, it is important that the health care staff have a sharp ear for recognizing potential obstacles to the awareness of patients in order to minimize such obstacles. The physicians then should:

• not narrowly focus on the treatment calendar

• not only deliver the results of each test taken but explain how it fits together with the entirety of all tests taken

• help these patients to "read between the lines" to make them aware of what probably is to come

• communicate better with these patients about the difficult questions of their approaching death and not wait for the patients to ask

• help these patients to become more aware of when their disease most probably is turning lethal

Finally, the health care staff could encourage the patients to have a more proactive attitude in their communication with their physician.

## Competing interests

The author(s) declare that they have no competing interests.

## Authors' contributions

GH was the philosophical and ethical supervisor of the study. He contributed to the conception and design of the study and has given substantial critique during the process of writing. GH has approved the final manuscript. LH has been the interviewer, data collector, analyser and writer.

## Pre-publication history

The pre-publication history for this paper can be accessed here:



## Supplementary Material

Additional file 1 Interview guide (to the first interview with each patient). Click here for file

Additional file 2 Interview guide (to remaining interviews). Click here for file
