# Supplementary material for: Information from physicians and retention of information by patients – Obstacles to the awareness of patients of progressing disease when life is near the end
Source: BMC Palliat Care. 2008 Feb 28;7:2. doi: 10.1186/1472-684X-7-2 (PMC2292688; doi:10.1186/1472-684X-7-2)
Supplement: Additional file 2 — Interview guide (to remaining interviews). [file 1472-684X-7-2-S2.doc]

**Additional file 2**

**Additional file 2 - Interview guide (to remaining interviews)**

First a short recapitulation of the previous interview and then questions like:

1) What has happened to you since we last met?

2) How are you today? What is worse? What is better?

3) What is there to tell about the treatment you are getting?

4) Any news in the information given to you?

5) What now?
